# Limitations of muscle biopsy in Pompe disease

**DOI:** 10.1186/1471-2474-14-S2-O5

**Published:** 2013-05-29

**Authors:** John Vissing

**Affiliations:** 1 Neuromuscular Research Unit, Department of Neurology, Rigshospitalet, University of Copenhagen, Copenhagen, Denmark

## 

The diagnosis of Pompe disease in children and adults can be challenging because of the heterogeneous clinical presentation and considerable overlap of signs and symptoms found in other neuromuscular diseases. This review evaluates the use of muscle biopsy and other methods for the diagnosis and differential diagnosis of late-onset Pompe disease. Muscle biopsy is commonly used as an early diagnostic tool in the evaluation of muscle disease. However, experience has shown that relying solely on visualizing a Periodic acid-Schiff-positive vacuolar myopathy to identify late-onset Pompe disease often leads to false-negative results, and subsequently delays diagnosis and treatment of the disorder. Serum creatine kinase can be normal or only mildly elevated in late-onset Pompe, so is also not very helpful alone to suggest the diagnosis, but may, in combination with proximal and axial weakness, raise the suspicion for Pompe disease. The optimal initial test for confirming or excluding Pompe disease is a simple, blood-based assay to measure the level of α-glucosidase activity. In particular, measurement of GAA activity in dried blood specimens is minimally invasive, quick, cost-effective, and reliable. Blood-based assays allow for the timely and accurate diagnosis of late-onset Pompe disease, and are likely to improve patient outcomes, as care standards, including enzyme replacement therapy, can be applied and complications may be anticipated and avoided. Increased awareness of the clinical phenotype of Pompe disease is essential in order to expedite diagnostic screening using blood-based, enzymatic assays.

